# A Case of COVID-19 With Memory Impairment and Delayed Presentation as Stroke

**DOI:** 10.7759/cureus.10025

**Published:** 2020-08-25

**Authors:** Alpana Garg, Amin Marji, Sachin Goyal, Rana Ismail

**Affiliations:** 1 Internal Medicine, Wayne State University Detroit Medical Center, Detroit, USA; 2 Neurology, Wayne State University Detroit Medical Center, Detroit, USA; 3 Gastroenterology, Wayne State University, Detroit, USA; 4 Clinical Research, Wayne State University, Detroit, USA

**Keywords:** covid-19, stroke, memory issues, sars-cov-2 (severe acute respiratory syndrome coronavirus -2), ischemic cva, stroke recrudescence, cycle threshold

## Abstract

The severe acute respiratory syndrome coronavirus 2 (SARS-CoV-2) is a respiratory pathogen with remarkable properties of multisystem involvement and numerous complications. The coronavirus disease of 2019 (COVID-19) presenting as stroke is becoming more common. Herein, we describe an unusual case of COVID-19 in a patient who initially presented with myalgia, fatigue, loss of taste and smell, and non-specific memory impairment. Two months after infection with SARS-CoV-2, the patient presented with acute onset of right-sided weakness, sensory loss, and worsening cognitive impairment. Reverse transcription-polymerase chain reaction was performed to detect the SARS-CoV-2 virus, and the results were positive at the time of initial infection as well as during the current admission. Neuroimaging suggested a subacute ischemic infarct in the middle cerebral artery. The patient was re-tested for SARS-CoV-2 and found to be positive, but the cycle threshold was high (40.4) along with a positive test for immunoglobulin-G (IgG) for SARS-CoV-2. This report highlights a unique case of stroke-related to COVID-19 infection in a middle-aged woman with otherwise mild symptomatic illness. The patient had a chronic ischemic stroke with delayed presentation two months after the initial symptomatic viral infection. This case underscores the importance of neurological assessment as well as timely evaluation of patients presenting with COVID-19 and any neurological concerns to recognize stroke as a complication of COVID-19 promptly.

## Introduction

Novel coronavirus disease 2019 (COVID-19) is associated with significant morbidity and mortality, with more than 19 million cases and 727,000 deaths worldwide as of August 8, 2020 [1]. The disease ranges from asymptomatic infection to severe illness, which varies among different patients for reasons that are currently unclear [2]. Stroke is one such complication that is being increasingly reported with COVID-19 and can have significant long-term effects on the individual and the caregivers [3-7]. The incidence of stroke as a complication in patients with COVID-19 was reported as 0.9% in a large study that included 3,556 hospitalized patients in New York [8]. The occurrence of stroke in COVID-19 is especially unique because patients in younger age groups can present with massive strokes without any underlying traditional risk factors for stroke [6]. Infection with severe acute respiratory syndrome coronavirus 2 (SARS-CoV-2) is associated with coagulopathy and thromboembolism, and this is particularly recognized as one of the risk factors for stroke [9,10].

## Case presentation

A 59-year-old white woman presented to the emergency department with acute right-sided weakness and memory loss. Six hours before the onset of her weakness, she went to sleep, and upon waking in the morning, she felt weakness in the right upper and lower extremities associated with tingling and numbness in her right foot. She also had an associated mild headache that began at approximately the same time after waking and was located mainly behind her right eye. She was experiencing memory deficits, such as difficulty remembering the names of her children and her work schedule for the day, as well as the names of supermarkets near her house, as noted by her family member. 

She was diagnosed with COVID-19 two months prior to the current admission when her symptoms included myalgia, loss of taste and smell, and fatigue for one week. She was not hospitalized at that time, and after viral detection, she observed home quarantine for two weeks. She reported feeling mental fogginess and experienced difficulty with memory and executive functioning after the viral illness. However, her memory deficits acutely worsened on the morning of presentation, and the right-sided weakness was new for her. 

She worked as a radiology technician at a local hospital. Her past medical history was significant for hypertension controlled on losartan and osteoarthritis, for which she took occasional ibuprofen. There was no history of stroke or any other neurological concerns in the past. She denied any history of blood clots, heart disease, or any features of autoimmune disease in the past. She underwent knee replacement for arthritis two years ago. She denied any history of smoking, alcohol, or any illicit drug use. Her family history was significant for Parkinson’s disease, which afflicted her mother. 

On physical exam, she was afebrile, her heart rate was 78 beats per minute, blood pressure was 140/80 mmHg, and respiratory rate was 18/minute. Her body mass index was 30.1 kg/m2. She was awake, alert, and oriented to time, place, and person. Her speech was fluent but non-purposeful with confabulation. She was displaying some evidence of word salad. When asked a question, she would also often sputter and go off on tangents, and it was hard to redirect her. 

The results of her cranial nerve exam were unremarkable except her visual field exam suggested superior quadrantanopia on the right side. Her motor exam showed 3/5 weakness on her right side in the lower extremity and normal strength elsewhere. Her sensory examination revealed diminished light touch in the dorsum of the right foot with pinprick, and vibration intact bilaterally. All the deep tendon reflexes were intact, and Babinski’s reflex was negative. Based on her history and exam findings, her National Institute of Health Stroke Scale score was 4 (1 for superior quadrantanopia, 1 for sensory, 1 for lower motor, and 1 for language). Her nasopharyngeal swab tested positive for novel SARS-CoV-2 reverse transcriptase-polymerase chain reaction (RT-PCR). Other laboratory findings were suggestive of lymphopenia, with an absolute lymphocyte count of 800 cells/mm3. The serum immunoglobulin-G (IgG) antibody test for SARS-CoV-2 was positive. The inflammatory markers were within normal range and are shown in Table 1.

**Table 1:** Laboratory results Abbreviations: dL, deciliter; EIA, enzyme-linked immunosorbent assay; fL, femtoliter; g, grams; HbA1c, glycated hemoglobin; K/CUMM, 1000/mm3; IgG, immunoglobulin G; L, liter; LDL, low-density lipoprotein; mg, milligram; mM, millimoles; ng, nanogram; OR, odds ratio; pg, picogram; RT-PCR, reverse transcription-polymerase chain reaction; S/CO, signal-to-cutoff ratio; SARS-CoV-2, severe acute respiratory syndrome coronavirus 2; U, units.

Test	Result	Test	Result
Sodium (mM/L, ref range 136-145)	141	Hemoglobin (g/dL, ref range13.3-17.1)	12.2
Potassium (mM/L, ref range 3.5-5.1)	4.6	Platelets (K/CUMM, ref range 150-450)	295
Chloride (mM/L, ref range 98-107)	107	Mean corpuscular volume (fL, ref range 81-98)	97.4
Bicarbonate (mM/L, ref range 21-31)	27	Mean corpuscular hemoglobin (pg, ref range 27.1-34.0)	31.4
Glucose (mg/dL, ref range 75-105)	108	Mean corpuscular hemoglobin concentration (%, ref range 32.6-35.4)	32.2
Blood urea nitrogen (mg/dL, ref range 7-25)	14	White cell count (K/CUMM ref range 3.5-10.6)	5
Creatinine (mg/dL, ref range 0.7-1.30)	0.7	Absolute lymphocyte count (K/CUMM, ref range 1.0-3.8)	0.8
Magnesium (mg/dL, ref range 1.6-3.0)	2.2	Prothrombin time (seconds, range 9.4-11.7)	9.7
Creatinine phosphokinase (U/L, ref range 30-223)	71	International normalized ratio	0.93
Lactate dehydrogenase (U/L, ref range 140-271)	197	Activated plasma thrombin time (seconds, range 23.1-33.1)	23.0
C-Reactive protein (mg/L, normal high < 5.0)		Syphilis EIA screen	< 0.10
D–Dimer (mg/L, normal high < 0.50)	0.30	HbA1c	5.4%
Ferritin (ng/mL, ref range 23.9-336.2)	58.9	Serum cholesterol (mg/dL, normal < 200)	145
Fibrinogen (mg/dL, ref range 186-466)		Triglycerides (mg/dL, normal < 150)	68
SARS-CoV-2 nasopharyngeal RT-PCR	Positive	Cholesterol-HDL (mg/dL, ref range 23-92)	61
SARS-COV2 IgG antibody (S/CO) < OR = 0.80 non-reactive for SARS-COV-2; IgG > 0.80 to < 1.00 S/CO = equivocal for SARS-COV2; IgG > or = 1.00 S/CO = reactive for SARS-COV-2 antibodies	3.98	Cholesterol-LDL (mg/dL, normal < 130)	70

**Figure 1:**
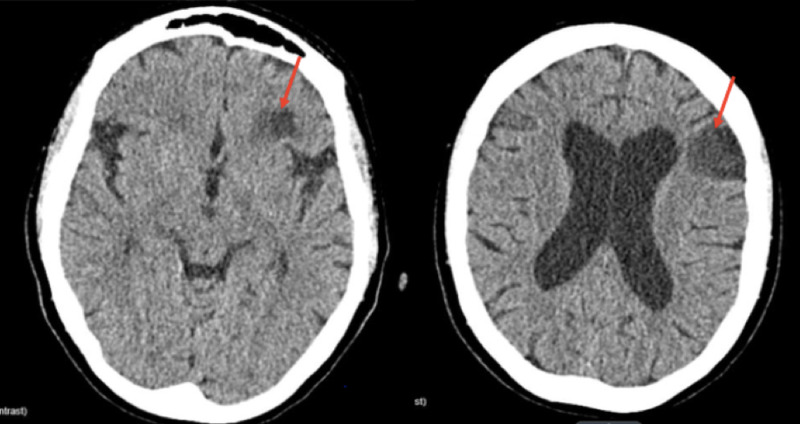
Noncontrast CT of the patient’s head showing hypo-density in the left frontal lobe (red arrows). Abbreviation: CT, computed tomography

Computed tomography (CT) of the head without contrast showed left frontal lobe hypodensity, as shown in Figure 1.

**Figure 2:**
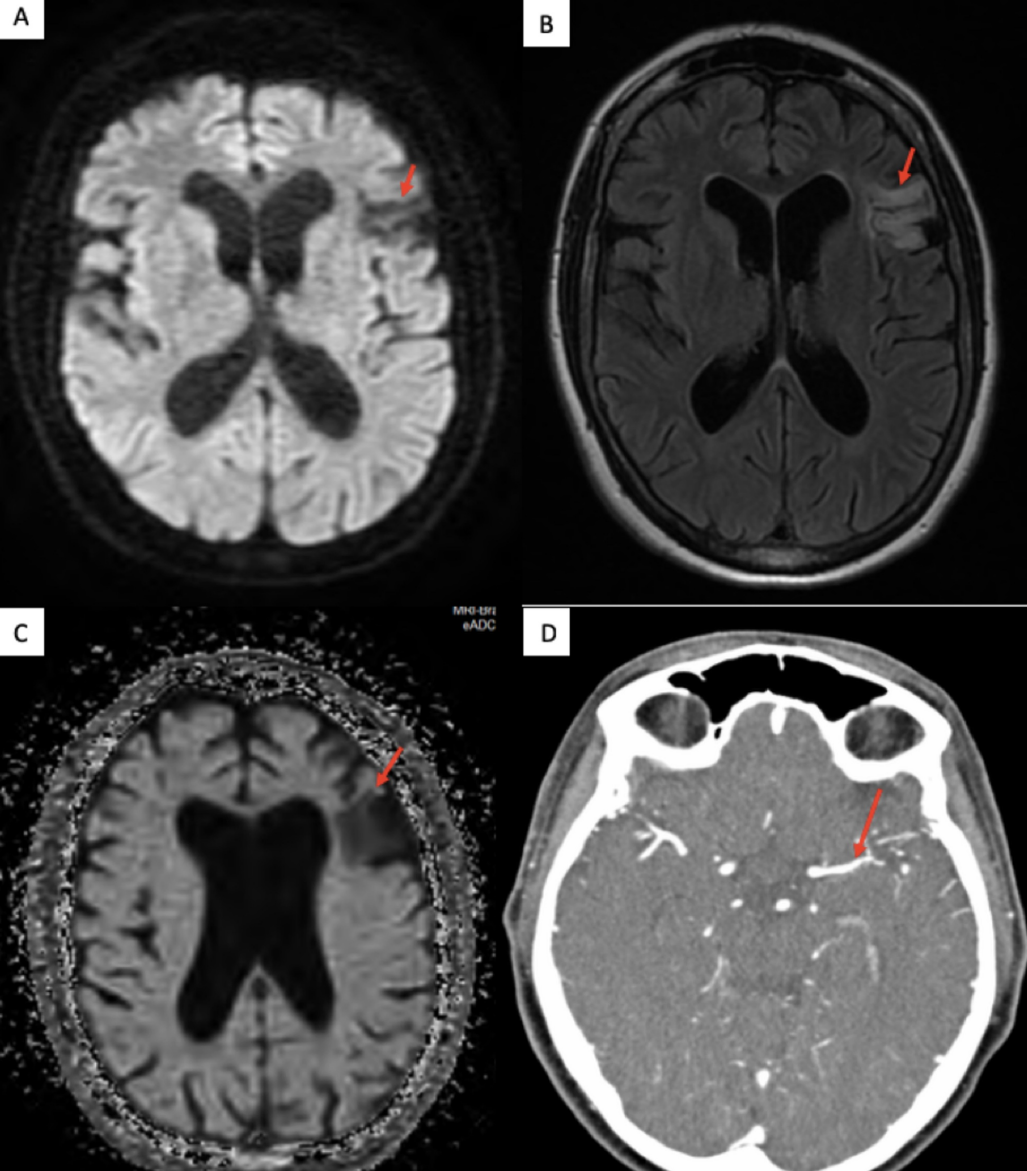
MRI of the brain showing (A) a hypo-intense signal in the left frontal lobe on diffusion-weighted imaging sequence, (B) a hyper-intense signal in the left frontal lobe on T2 flair sequence, (C) a hypo-intense signal on apparent diffusion coefficient sequence, and (D) a mild narrowing left MCA on CT angiography. Abbreviations: MRI, magnetic resonance imaging; MCA, middle cerebral artery; CT, computed tomography

We compared the current CT scan to a previous CT scan of her head (which was done two years ago) that showed no lesion and was reported normal. CT angiography of the head, brain, and neck with contrast revealed minimal calcification at the left common carotid bifurcation without stenosis. The mid-cervical segment of the bilateral internal carotid arteries was tortuous (Figure 2D).

**Figure 3:**
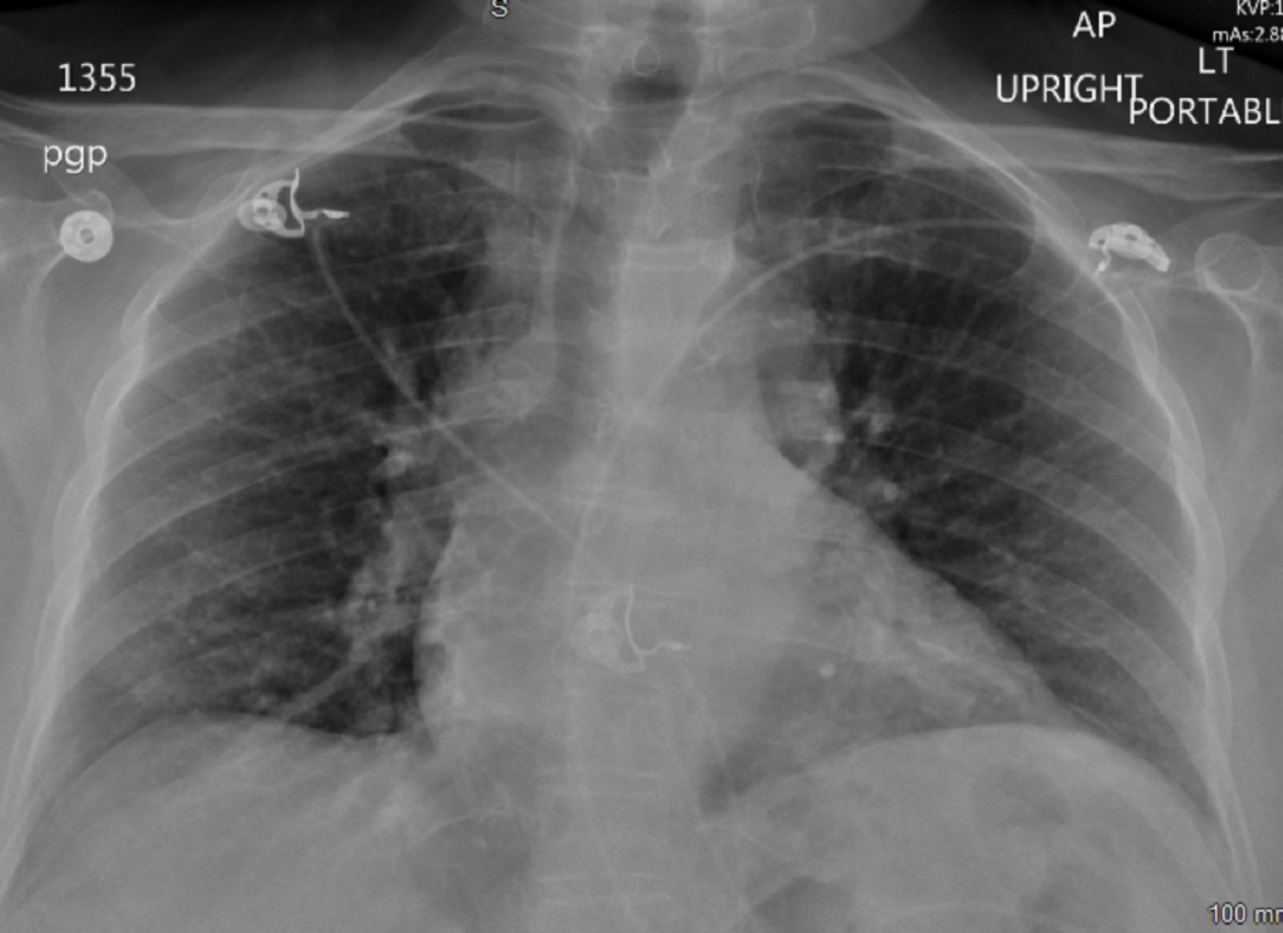
Chest X-ray showing no significant abnormalities and reported as normal

There was mild diffuse narrowing in the distal M1 and M2 segment of the left middle cerebral artery (MCA). There was no proximal branch occlusion or aneurysm. CT of the head/brain with perfusion (CTP) indicated matched perfusion defect in the left frontal lobe, suggesting core infarct. There was no definitive evidence of any mismatch perfusion defect to suggest reversible ischemic penumbra. Magnetic resonance imaging of the brain with contrast revealed hyperintense signal abnormalities in the left frontal lobe involving the operculum and insula, which were suggestive of an old infarct in the left MCA territory. There was mild ex vacuo dilatation of the left lateral ventricle (Figure 2A, 2B, 2C). A two-dimensional echocardiogram did not show any evidence of thrombus, and electrocardiogram, along with 48 hours of telemetry, did not reveal any arrhythmias. Her chest X-ray was reported normal (Figure 3).

The patient did not qualify for tissue plasminogen activator administration, considering the delay in presentation and the time frame allotted for it in the case of ischemic stroke [11]. With the new onset of neurological symptoms and signs, she was admitted under the neurology service for close monitoring and frequent neuro-checks. During her hospital stay, her weakness appeared to improve mildly, but she continued to experience cognitive impairment. On her Montreal Cognitive Assessment screening test, she scored 27/30 (1 point for loss in visuospatial/executive function, 1 point for language, and 1 point for delayed recall). She was started on aspirin, clopidogrel, and high-intensity statin; dual anti-platelet agents were prescribed for three weeks, followed by lifelong aspirin. Although the role of therapeutic anticoagulation is being increasingly investigated for COVID-19 patients, the regimen was not initiated in this patient based on D-dimer and other coagulation test results that were normal. The discharge plan consisted of home physical therapy, follow-up appointment with a stroke specialist and an outpatient thrombophilia work-up after a few months.

## Discussion

We present an interesting case of COVID-19 infection in a middle-aged woman presenting with stroke, manifesting as a cognitive impairment that was undetected during her initial COVID-19 infection two months earlier. The patient exhibited a mild symptomatic viral illness with non-specific neurological complaints such as loss of olfaction, taste, and fatigue, but began having some degree of memory impairment without any other neurological symptoms. However, she presented two months later with worsening cognitive impairment, as well as sensory deficits and new right-sided weakness. Apart from hypertension, which was controlled by medication, she did not have any underlying vascular risk factors for stroke. COVID-19 infection is known to increase the risk of stroke and various thromboembolic complications [12]. 

Initial reports suggest that COVID-19 can present with new-onset neurological impairment and even massive stroke, especially in young patients. The majority of neurological complications, especially stroke, occurred in patients who were critically ill [6,7]. Hypertension was the most common vascular risk factor, and stroke in the region of the brain supplied by the MCA was associated with COVID-19 patients in the case series reported by Sierra-Hudalo et al. [13]. There was no evidence of a hypercoagulable state in the present case, and the patient was never critically ill during the viral illness, as evidenced by a normal D-dimer and other coagulation studies. Besides the hyper-coagulable state, direct neuronal injury, vasculitis, or any other pathophysiological mechanisms of stroke in SARS-CoV-2 infection are yet to be identified [14]. Further investigation is needed to clarify the role of early identification of patients at risk of thromboembolic complications and having a lower threshold for therapeutic anticoagulation in such patients. 

Nasopharyngeal swab tests indicated that the patient continued to test positive for SARS-CoV-2 two months after the initial test. Viral shedding with continued positive RT-PCR results for SARS-CoV-2 has been reported at eight weeks, even in patients with mild illness [15,16]. Repeat positive tests several weeks post-infection seemed puzzling, given the patient’s new onset of neurological manifestations. This could be indicative of ongoing active infection, reinfection, or recovering illness. The cycle threshold (Ct) of the SARS-CoV-2 ribonucleic acid (RNA) PCR test conducted during the current presentation was 40.4. In a recent study, a reported Ct value > 34 was associated with low viable viral load with no growth of SARS-CoV-2 on viral cultures [17]. Because viral RNA may shed for much longer than the infectious virus, it was more plausible to assume that our patient was in the recovery phase of the illness. Also, the presence of IgG antibody was suggestive of antibody response to the recent COVID-19 infection. However, it is unclear at present if viral infectivity should be determined based on continued positive PCR results for SARS-CoV-2, and further studies are required for confirmation of this on a larger scale [18]. 

Interestingly, the neuroimaging was suggestive of an old infarct with new-onset symptoms, which may possibly be related to post-stroke recrudescence (PSR). This has been previously very well described in a cohort study that included 153 patients, of whom 112 patients (73%) exhibited re-emergence of previous stroke-related deficits involving white matter tracts and MCA territory [19]. Stress, metabolic derangements, and infections are some of the risk factors for PSR. PSR is possible even with a remote history of stroke. However, it is emphasized that the patient should be evaluated based on clinical judgment when presenting with new neurological events.

## Conclusions

This report highlights a unique case of late-onset stroke symptoms related to SARS-CoV-2 infection. The patient had a chronic ischemic stroke in the setting of a recent SARS-CoV-2 infection manifesting as memory impairment. The acute neurological deficits two months after infection may be related to stroke recrudescence during the recovery from SARS-CoV-2 infection. This case elucidates the importance of neurological assessment as well as timely evaluation of patients presenting with COVID-19 and any neurological complaints. Further studies are required to determine the risk factors, pathophysiology, treatment, and prevention modalities for acute strokes related to SARS-CoV-2 infection.
